# Enhancing Thermal Conductivity of Polyvinylidene Fluoride Composites by Carbon Fiber: Length Effect of the Filler

**DOI:** 10.3390/polym14214599

**Published:** 2022-10-29

**Authors:** Guoqing Yi, Jingliang Li, Luke C. Henderson, Weiwei Lei, Lian Du, Shuaifei Zhao

**Affiliations:** 1 Institute for Frontier Materials, Deakin University, Geelong, VIC 3216, Australia; 2 Nanjing CAS Bidun Newmem Technology Co., Ltd., Nanjing 210061, China

**Keywords:** polyvinylidene fluoride, carbon fiber, polymer composites, thermal conductivity

## Abstract

Thermally conductive polyvinylidene fluoride (PVDF) composites were prepared by incorporating carbon fibers (CFs) with different lengths (286.6 ± 7.1 and 150.0 ± 2.3 µm) via cold pressing, followed by sintering. The length effects of the CF on the thermal conductivity, polymer crystallization behaviors, and mechanical properties of the PVDF composites were studied. The through-plane thermal conductivity of the PVDF composites increased significantly with the rise in CF loadings. The highest thermal conductivity of 2.89 W/(m∙K) was achieved for the PVDF composites containing 40 wt.% shorter CFs, ~17 times higher than that of the pure PVDF (~0.17 W/(m∙K)). The shorter CFs had more pronounced thermal conductive enhancement effects than the original longer CFs at higher filler loadings. CFs increased the storage modulus and the glass transition temperature of the PVDF. This work provides a new way to develop thermally conductive, mechanically, and chemically stable polymer composites by introducing CFs with different lengths.

## 1. Introduction

Polymers have been extensively used in daily life and industries, such as food packaging [1], membrane separation [2], agriculture [3], and medicine [4], due to their light weight, low production cost, robust mechanical properties, good chemical stabilities, etc. [5,6,7]. They have been widely used as corrosion resistant materials and electrical insulation, and they have some utilities in heat dissipation systems [8,9,10]. However, most polymers have low thermal conductivities, typically below 0.5 W/(m∙K) [11], which significantly limits their applications in thermal management systems, such as polymer heat exchangers and thermal interfacial materials [12,13,14]. Therefore, enhancing the thermal conductivity of polymers is of great significance [15].

To improve the thermal conductivity of polymers, it is popular to construct polymer composites by adding thermally conductive fillers into polymer matrices. Ding et al. [16] synthesized silicon rubber composites with vertically-oriented carbon fibers (CFs) by a cast molding method. The results showed that the maximum thermal conductivity was 4.72 W/(m∙K), with an enhancement of 2045.5% at a filler loading of 9 vol.%. Wang et al. [17] fabricated ethylene-vinyl acetate/carbon nanotubes@boron nitride (CNTs@BN) composites by liquid blending and hot pressing and achieved a high thermal conductivity of 7.84 W/(m∙K) with 30% of CNTs@BN hybrids. Hou et al. [18] prepared CF/polydimethylsiloxane (PDMS) composites via constructing vertically-oriented CF structures and achieved a thermal conductivity of 6.04 W/(m∙K) at a relatively low filler loading (12.8 vol.%).

Carbon-based materials (e.g., graphene and carbon nanotubes) have played an important role in various applications, such as conductive composites and energy storage, due to their unique physical, chemical, thermal, and electrical properties [19,20,21]. CFs have attracted attention as reinforcement fillers for polymer matrices because of their light weight, high mechanical strength, excellent thermal stability, and high thermal conductivity [22,23,24,25]. CFs can be either added into a polymer matrix directly to construct a polymer composite or treated by surface modifications to improve their interfacial compatibility with a polymer matrix [26,27,28]. CFs can enhance the thermal and mechanical properties of the polymers, as well as reduce the density of the materials [18]. In addition, CFs exhibit high thermal conductivity, from 530 to 1100 W/(m∙K), along the axial direction [29]. Therefore, CFs are good candidates for thermal conductivity enhancement of polymers.

Polyvinylidene fluoride (PVDF) has been widely used in the chemical, water treatment, and other industries [30,31,32] because of its excellent resistance to chemical corrosion, good thermal stability, mechanical properties, and a wide working temperature range [33,34]. However, the intrinsic thermal conductivity of PVDF at room temperature is low (~0.19 W/(m∙K)) [29]. Many efforts have been made to enhance the thermal conductivity of PVDF. Cao et al. [35] constructed PVDF composites with two dimensional graphene sheets by solution blending and compression molding. The maximum thermal conductivity of the PVDF composite was 10 times higher than that of the pure PVDF. Guo et al. [36] prepared aligned polyaniline (PANI)/PVDF composite membranes assisted by an electric field. The highest thermal conductivity (~0.33 W/(m∙K)) of the aligned membrane with a PANI loading of 50 wt.% was nearly 85% greater than that of the neat PVDF membrane. Despite the fact that the thermal conductivities improved in these studies, incorporating fillers to improve the thermal conductivity of PVDF remains a challenge, since the improvement is still far from satisfactory.

Apart from the intrinsic properties of fillers and polymers, the size of fillers is also important to the final properties of the polymer composites. For thermal conductivity, the aspect ratio is an important parameter: a high aspect ratio facilitates the formation of thermally conductive networks at lower volume fractions because of the lower percolation threshold [37,38,39]. However, there are fewer systematic studies on the effect of CF sizes on the thermal conductivity of PVDF-based composites.

In this study, we investigated the effect of the filler length on the thermal and mechanical properties of polymer composites using PVDF as the polymer matrix and CFs as the filler. For comparison, two series of PVDF-based composites were prepared by adding CFs possessing different lengths to the PVDF matrix. Considering the potential application of the composites in polymer heat exchangers, the thermal and mechanical properties of the PVDF/CF composites, including the thermal conductivity, polymer crystallization behaviors, thermal stability, dynamic mechanical behaviors, and mechanical properties, were systematically investigated. The chemical stabilities of the PVDF/CF composites were also investigated by long-term contact with acid and alkaline solutions.

## 2. Experimental

### 2.1. Materials

PVDF powder was purchased from Sigma-Aldrich Pty Ltd. (an affiliate of Merck KGaA, Darmstadt, Germany) (average M_w_ ~534,000, Sydney, Australia). The pristine milled CF (K223HM, PITCH 90t, fiber diameter of 11 µm, thermal conductivity of 550 W/(m∙K)), without sizing agents, was provided by Mitsubishi Chemical Cooperation (Tokyo, Japan). Other chemicals included ethanal (undenatured 100% AR, Chem-supply, Gillman, Australia), sulfuric acid (96%, Sigma-Aldrich, Sydney, Australia), and sodium hydroxide (≥98%, Sigma-Aldrich, Sydney, Australia).

### 2.2. Size-Down of CFs

A certain amount of CF was mixed with deionized water. The mixture was sonicated by a horn-based sonicator (Q700 Sonicator, QSonica Sonicators, Newtown, CT, USA) for 1 h at an amplitude of 55 with 3 s pulse on and 1 s pulse off. After sonication, the CFs were filtered under vacuum, and the disk of the wet CFs was removed from the filtration paper. The wet CFs then were dried under vacuum at 105 °C for 24 h. The sonicated CFs with reduced sizes were marked as SCF (small-sized CF). The original CF was marked as CF.

### 2.3. Preparation of PVDF/CF Composites

**Figure 1:**
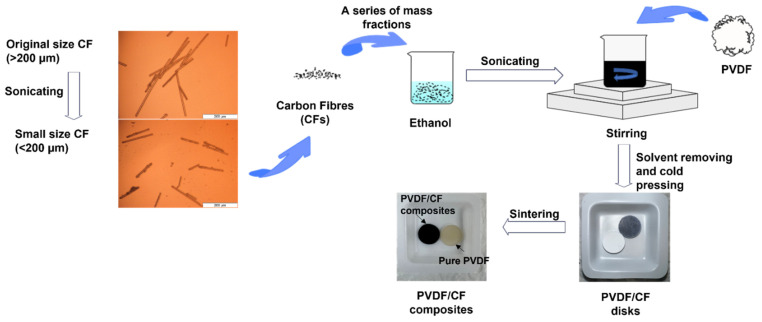
Preparation procedure of the PVDF/CF composites.

The preparation procedure of the PVDF/CF composites is illustrated in Figure 1. A certain amount of CF was dispersed in ethanol and sonicated for 30 min. Meanwhile, a certain amount of PVDF powder was mixed with ethanol and stirred at high-speed shear for 30 min. Then, these two ethanol dispersions were mixed and stirred for 1 h, followed by heating at 60 °C until the mixture turned to a slurry. The slurry was then left on the hot plate and evaporated to dryness after the hot plate was turned off. The mixture was then placed in a ventilated oven at 105 °C for 12 h. The dried mixture was then kept in a vacuum oven at 60 °C for 2 h before use.

The prepared mixture was then pressed to small disks by a hydraulic press machine (Atlas Autotouch, Specac, Orpington, UK) with a loading of 8 tons and holding time of 3 min. The disk (diameter 12.9 mm) was polished to 12.7 mm for further tests. The thickness of each disk was 1.5–2 mm. The disk was then sintered in a tube furnace (STF16/610 Tube Furnace, Carbolite, Hope Valley, UK) at 160–170 °C, with a heating rate of 2 °C/min, and kept under a nitrogen atmosphere for 30 min. After cooling, the disk became hard and had a plastic texture, suggesting the formation of the PVDF/CF composites.

The prepared composites were summarized in Table 1. The maximum filler loading was 40 wt.%, as further higher filler loadings led to poor mechanical properties [40]. Therefore, to better investigate the composite properties for polymer heat exchangers, the loadings below 40 wt.% were selected.

### 2.4. Materials Characterization

Scanning electron microscopy (SEM, Supra 55VP, Zeiss, Oberkochen, Germany) was used to characterize the fractured surface morphology of the PVDF/CF composite. Before imaging, the composite was fractured in liquid nitrogen and then fixed by conductive adhesive tapes for platinum coating.

The thermal diffusivity (α) of the composites was measured by a laser flash analyzer (LFA 457, NETZSCH, Selb, Germany). To avoid influence of dust on the sample surface, all the samples were cleaned with ethanol prior to each measurement and coated by graphite for a better heat adsorption. The measurement was repeated three times, with a time interval of 3 min at each temperature, and each data point was kept for 3 s. All measurements were undertaken at an applied voltage of 1.5 V. The temperatures of 25, 50, and 75 °C were selected for the measurement, considering the potential practical applications of the polymer composites for thermal management. The through-plane thermal conductivity (*λ*) was calculated by the equation *λ*(*T*) = α(*T*) × *C_p_*(*T*) × *ρ*(*T*), where α is the thermal diffusivity (mm^2^/s), *C_p_* is the specific heat capacity (J/(kg·K)), and *ρ* is the density (kg/m^3^). These parameters are the functions of temperature (*T*).

The melting and crystallization behaviors of the PVDF/CF composites were investigated by differential scanning calorimetry (DSC) (TA Q200, TA Instruments, New Castle, DE, USA). The mass of each sample was 5–10 mg. Firstly, the samples were heated from room temperature to 200 °C at a heating rate of 30 °C/min, and then held at 200 °C for 5 min to erase thermal history, followed by cooling to 20 °C at a cooling rate of 10 °C/min. After cooling, the samples were reheated to 200 °C at a heating rate of 10 °C/min. For each loading, three samples were tested. All the measurements were performed in a continuous nitrogen flow.

The crystallinity degree (*X_c_*) of the PVDF matrix is calculated by:

(1) $$X_{c}=\frac{\Delta H_{m}}{\emptyset \times \Delta H_{m}^{0}}\times 100\%\;$$

where $\Delta H_{m}$ (J/g) is the fusion enthalpy of the samples from the DSC heating scan. $\Delta H_{m}^{0}$ is the fusion enthalpy of the completely crystalline PVDF (104.6 J/g [41]). ∅ is the relative mass fraction of the PVDF in the composite.

A Fourier transform infrared spectroscopy (FTIR) spectrometer (Bruker, Bremen, Germany) with attenuated total reflectance (ATR-FTIR) was performed to evaluate the crystalline structure of the PVDF in the composites and the surface functional groups of the PVDF/CF composites treated by acid and base, respectively. The resolution was set to 4 cm^−1^, and the wavenumber ranged from 600 to 4000 cm^−1^.

To demonstrate the crystalline structure change of the PVDF matrix, Equations (2) and (3) were used to calculate the relative proportions of α- and β-phase [33].

(2) F(α)=AαAα+0.8Aβ 

(3) F(β)=AβAβ+1.26 Aα 

The dynamic mechanical behaviors of the samples were characterized by a dynamic mechanical analyzer (TA Q800, TA Instruments, New Castle, DE, USA) in the tension mode. The frequency was set to 1 Hz, and the temperature range was from −50 to 110 °C at a heating rate of 3 °C/min.

Thermogravimetric analysis (TGA) was conducted employing TA Q50 (TA Instruments, New Castle, DE, USA) instrument, in which the sample was heated from room temperature to 600 °C at a heating rate of 10 °C/min in a nitrogen atmosphere. For each loading, three samples were tested.

Tensile tests were performed on a tensile strength testing machine (Instron 5567, Instron, Norwood, MA, USA). The samples (10 mm × 100 mm) were slowly pulled at a constant rate of 10 mm/min until they broke.

## 3. Results and Discussion

### 3.1. Length Change of CFs before and after Treatment

**Figure 2:**
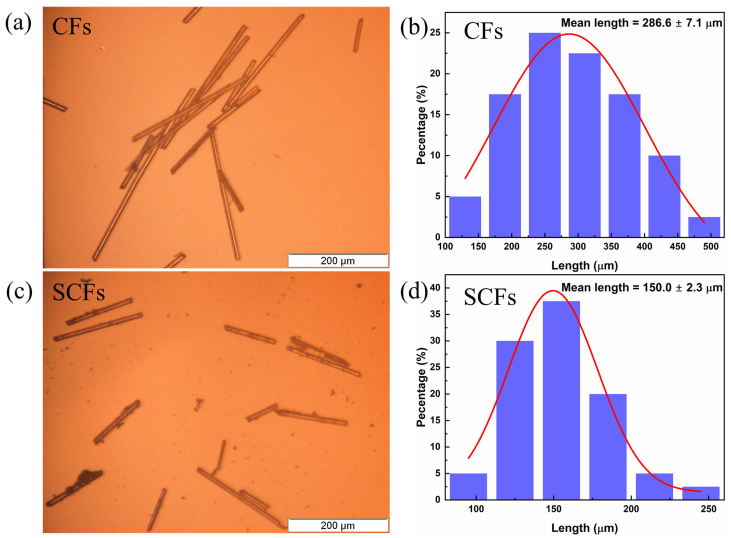
Microscopic images of the CFs and SCFs (**a**,**c**) and their length distributions with Gaussian fittings (**b**,**d**).

The microscopic images of the CFs (Figure 2a) and SCFs (Figure 2c) and their length distributions with Gaussian fittings (Figure 2b,d) are shown below. After sonicating, the lengths of the CFs changed significantly. Most of the original CFs were longer than 200 µm, and the mean length was 286.6 ± 7.1 µm (Figure 2b). However, the CF length reduced obviously after sonication. In Figure 2d, most of the SCFs were shorter than 200 µm, and the mean length was 150.0 ± 2.3 µm.

### 3.2. Surface Morphology of the PVDF Composite

**Figure 3:**
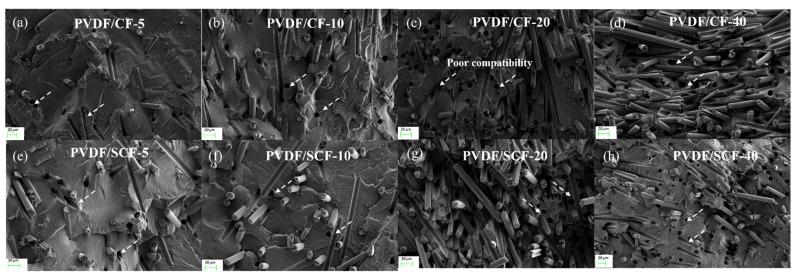
SEM images of the PVDF composites: (**a**–**d**) PVDF/CF; and (**e**–**h**) PVDF/SCF composite series. (Scale bar in each image: 20 µm).

The fractured surface morphologies of the PVDF/CF and PVDF/SCF composites are shown in Figure 3. For the PVDF/CF composites (Figure 3a–d), the CFs dispersed in the PVDF matrix moderately homogeneously at low CF loadings, while with increasing the CF loading, the dispersion of the CFs became poorer, and there was slight aggregation as the PVDF matrix exposing at the cross-sections of the composites significantly reduced. At low CF loadings (i.e., 5 wt.%, Figure 3a), the thermal conductive networks were built, thus increasing the content of the CFs led to denser CF network structures in the polymer matrix (i.e., 20 and 40 wt.%, Figure 3c,d). However, the poor compatibility between the CFs and PVDF matrix is the dominant issue. There were more voids in these composite systems (as indicated in white dash arrows in Figure 3), suggesting that the previously embedded CFs dropped off from the PVDF matrix, while the CFs dropped off from the PVDF matrix more easily with increasing CF loadings. This indicates that the compatibility between the CFs and PVDF matrix was poor, and adding more CFs led to poorer compatibility.

For the PVDF/SCF composites (Figure 3e,f), the morphologies were similar to the PVDF/CF composites. However, at high content loadings (i.e., 40 wt.%, Figure 3h), the dispersion of the SCFs was slightly more uniform than that of the CFs into the PVDF matrix, and the aggregation was not significant. In addition, the compatibility of the SCFs was poorer than that of the CFs, since there were fewer adhesion areas between the SCFs and PVDF matrix, and the chance of the SCFs dropping off from the PVDF matrix was larger than that of the CFs at the same loadings.

### 3.3. Thermal Conductivity

**Figure 4:**
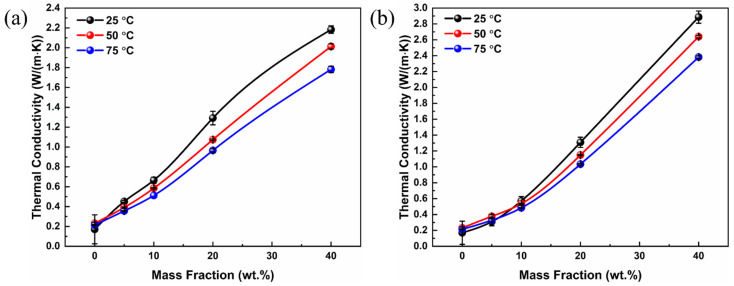
Variations of the thermal conductivity of PVDF/CF composites at different temperatures versus the content of CFs: (**a**) PVDF/CF and (**b**) PVDF/SCF composite series.

The through-plane thermal conductivities of the PVDF composites with various CF loadings at different temperatures are shown in Figure 4. Considering the possible working conditions of polymer heat exchangers (e.g., 50–80 °C [42]), three different temperatures (i.e., 25, 50, and 75 °C) were selected to measure the thermal conductivity of the composites. At 25 °C, the thermal conductivity of the pure PVDF sample was ~0.17 W/(m∙K), consistent with the reported thermal conductivity of PVDF [43]. For both the PVDF/CF and PVDF/SCF composites, the thermal conductivity increased with the rise in CF loadings. Taking the PVDF/CF composite series at 25 °C as examples (Figure 4a), the highest thermal conductivity of the PVDF/CF composites was 2.18 W/(m∙K), nearly 13 times larger than that of the pure PVDF when the CF loading was 40 wt.%. In addition, the thermal conductivity enhancement was less significant when the CF loading was below 10 wt.%. However, with further increasing CF loading, the enhancement increases obviously from 289% to 1178%. Similarly, for the PVDF/SCF composites, the highest thermal conductivity was 2.89 W/(m∙K), with the enhancement of 1589% at the CF loading of 40 wt.%.

**Figure 5:**
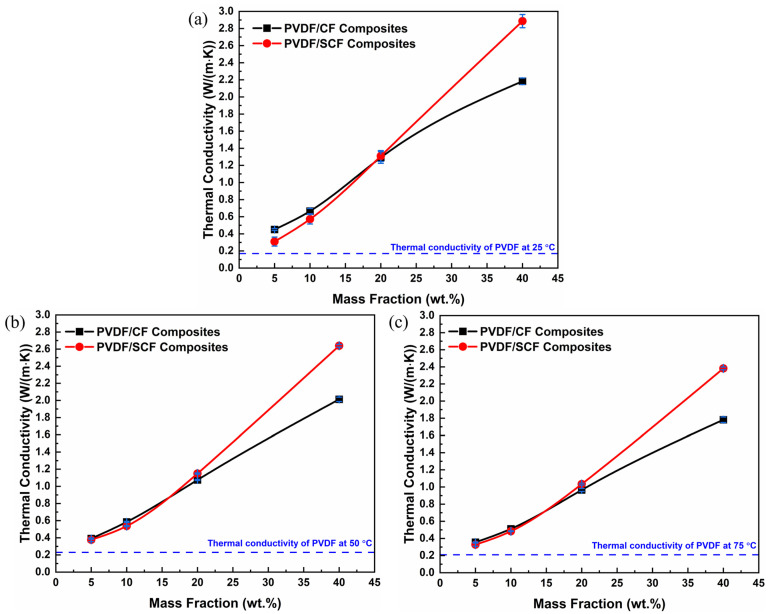
Thermal conductivities of the PVDF/CF and PVDF/SCF composites at (**a**) 25, (**b**) 50, and (**c**) 75 °C, respectively.

As shown in Figure 5a, the highest thermal conductivity of the PVDF/SCF composite (2.89 W/(m∙K)) was larger than that of the PVDF/CF composite (2.18 W/(m∙K)) at 25 °C. When increasing the measurement temperature, the difference in thermal conductivity between the PVDF/SCF and PVDF/CF composites became larger. The filler loading of approximately 20 wt.% was a critical value, above which the thermal conductivity of the PVDF/SCF composite was higher than that of the PVDF/CF composite and below which the thermal conductivity of the PVDF/SCF composite was slightly lower. At low CF loadings, the likelihood of longer CFs physically contacting with each other to construct CF networks was significantly higher. The connected CF networks were beneficial for phonon transport. Therefore, the thermal conductivity of the PVDF/CF composite was larger than that of the PVDF/SCF composite at lower filler loadings. As the CF loading increased, the dispersion of longer CFs into the PVDF matrix becomes difficult, due to aggregation. The poor dispersion and aggregation of CFs hinder phonon transport, reducing thermal transport properties. Although the compatibility is still poor between the shorter CFs and PVDF matrix, the higher filler loadings contribute to the increased likelihood for the small-sized CFs contacting each other. Meanwhile, small-sized CFs had better dispersion than the original CFs and built the networks better at higher filler loadings. As a result, the PVDF/SCF composites showed higher thermal conductivity values than the PVDF/CF composites at higher CF loadings.

### 3.4. Crystallization Behavior of the Polymer

The crystal structure of semi-crystalline polymer matrix has a significant influence on the thermal conductivity of polymer composites [44,45,46]. Furthermore, the nucleation effect of CFs influences the crystallization of semi-crystalline polymers [47]. PVDF is a typical semi-crystalline polymer and has five crystalline forms, including α, β, γ, δ, and ε forms [48]. Hence, it is worth investigating whether the increased thermal conductivity of the PVDF composites is associated with the changed crystal structure of the PVDF matrix or the addition of CFs at either length scales. We investigated the relationship of the microstructure of these two composite systems.

**Figure 6:**
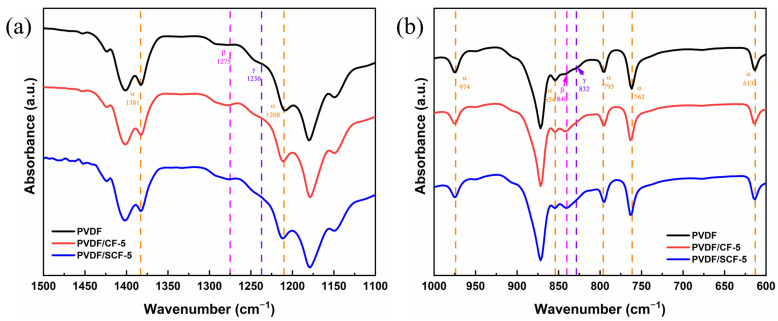
ATR-FTIR spectra of the pure PVDF, PVDF/CF-5, and PVDF/SCF-5 in different wavenumber ranges: (**a**) 1500–1100 cm^−1^ and (**b**) 1000–600 cm^−1^.

Pure PVDF, PVDF/CF-5, and PVDF/SCF-5 were selected as representative samples for crystal structure analysis of the PVDF matrix. The pure PVDF samples showed absorption bands at wavenumbers of 762, 795, 854, 974, 1208, and 1381 cm^−1^ (Figure 6), which are characterized as α-phase PVDF [49]. The two absorption bands at 832 and 1279 cm^−1^ are attributed to characteristic peaks of β-phase PVDF [49]. However, the PVDF/CF-5 showed a slightly stronger absorption band at 832 cm^−1^, which indicates the altered relative proportions of the α- and β-phase PVDF. Adding CFs into the PVDF matrix is favorable for the formation of the β-phase. Therefore, the structure of the PVDF within the composite was influenced. To clearly present the phase change of the PVDF, the relative proportions of the α- and β-phase have been calculated by Equations 2 and 3. The absorbance intensities of the α-phase bands at 762 cm^−1^ and the β-phase bands at 840 cm^−1^ were used to determine the percentages, as displayed in Table 2. Obviously, the proportion of the α-phase PVDF increased after adding CFs, in particular, small-sized CFs, into the PVDF matrix.

**Figure 7:**
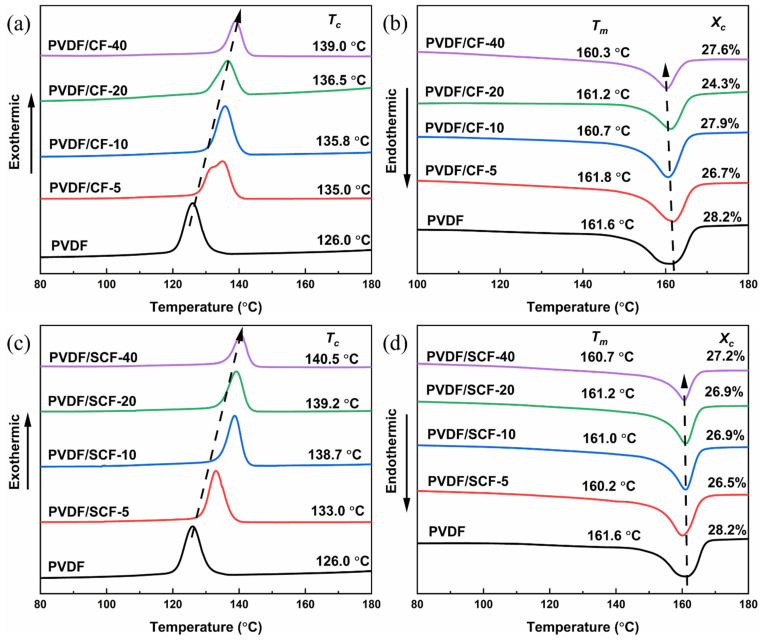
DSC cooling (**a**,**c**) and heating (**b**,**d**) curves of the samples.

To further explore the relationship between the crystalline structure of the PVDF and the thermal conductivity of the PVDF composites, we studied the melting and crystallization behaviors of the PVDF composites by DSC. The DSC heating and cooling curves of the samples were plotted, and the *X_c_* was calculated (Figure 7).

As shown in Figure 7a,c, the CFs showed a significant impact on the crystallization behavior of the PVDF. The crystallization temperature (*T_c_*) increased with an increase of the CF contents. The pure PVDF showed crystallization peak at around 126.0 °C. All the PVDF composites with original size and small-sized CFs had higher *T_c_* values than that of the pure PVDF, while small-sized CFs increased the *T_c_* value of the PVDF composites more significantly. This demonstrates that the heterogeneous nucleation effect of the CFs facilitated the crystallization of the PVDF matrix, with the small-sized CFs showing a more drastic effect. Specifically, there was a double exothermic peak for the PVDF/CF-5, which indicates that 5 wt.% of the original size CFs prompts an obvious variation in the crystallization behavior of the PVDF matrix. The first exothermic peak was primarily associated with the isolated CF-induced crystallization, and the second peak, at a higher temperature of 135.0 °C, was attributed to the connected CF-induced crystallization with more nucleation sites for the PVDF crystallization [50]. At relatively high CF loadings (i.e., 10, 20, and 40 wt.%), a single exothermic peak presented after a dense CF network structure formed throughout the composite systems. Moreover, the PVDF/CF composites still showed a lower *T_c_*, compared to the PVDF/SCF composites at the same filler loadings. This is because the original size CFs prevented the nucleation and growth of the PVDF crystallites on the surface of the CFs, whereas small-sized CFs had a stronger nucleation-promoting effect.

From Figure 7b,d, the pure PVDF showed a broad endothermic peak with a melting point (*T_m_*) of 161.6 °C, and the *X_c_* was 28.2%. For the PVDF/CF and PVDF/SCF composites, their *T_m_* values slightly declined, compared with that of the pure PVDF. However, the decrease was relatively small, within 2 °C. All the PVDF composites had lower *X_c_* values than that of the pure PVDF. All the samples showed similar *T_m_* and *X_c_*. It indicates that the CFs did not significantly affect the crystalline structure of the PVDF matrix in these composite systems. The explanation is that the adhesion between the CFs and the PVDF matrix was a weak physical interaction, which indicates that only a little energy was required to break the interaction. Therefore, the melting temperature of the composites was almost the same as that of the pure PVDF. In addition, the *T_m_* was proportional to the thickness of the lamella [51]. A higher *T_m_* suggests a thicker lamellar. Hence, the similar values of the *T_m_* in different composites suggest that all the composites had similar lamellar thickness.

In short, no obvious variations in the crystalline structure between the PVDF/CF and PVDF/SCF composites were observed. Although there was a conversion from α- to β-phase PVDF after adding the CFs into the PVDF matrix, the phase conversion was related to the electrical characteristics of the PVDF. All samples showed consistent crystallinity and lamellar thickness, although slightly smaller than that of the pure PVDF.

### 3.5. Thermal Stability Analysis of PVDF Composites

**Figure 8:**
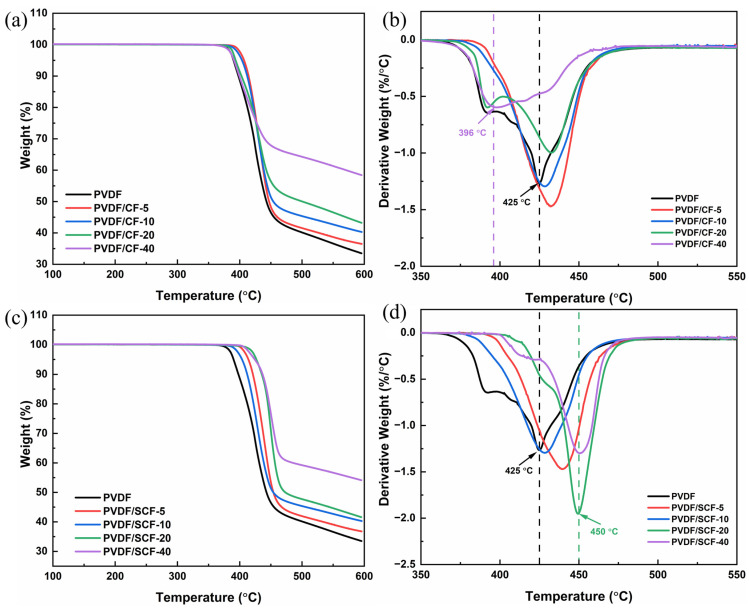
Thermal stability analysis of the pure PVDF and PVDF composites (**a**,**c**): TGA curves of the PVDF/CF and PVDF/SCF composite series, (**b**,**d**): DTG curves of the PVDF/CF and PVDF/SCF composite series.

The thermal stabilities of the pure PVDF and the composites were assessed via TGA. Figure 8a,c show the TGA curves for the pure PVDF and PVDF composites. Thermal decomposition of the pure PVDF and PVDF composites occurred in the temperature range of 390–440 °C, and the degradation profiles of all samples were similar. This is because the interaction between the fillers and the PVDF matrix was weak, and the decomposition temperature of the CFs was much higher than the ranges of 390–440 °C. Therefore, the decomposition was mainly attributed to the PVDF matrix. This suggests that adding CFs into the PVDF matrix did not significantly change the degradation mechanism of the PVDF matrix [52]. The weight loss, within the temperatures of 390–440 °C, was the potential loss of molecules, which indicates that the PVDF matrix started to deteriorate.

The derivative thermogravimetry (DTG) curve shows the peak temperature where the maximum weight loss rate was reached. The corresponding DTG curves of the samples are presented in Figure 8b,d. Table 3 lists the temperature for 5% weight loss (*T*_5%_), 10% weight loss (*T*_10%_), and maximum weight loss (*T_max_*). From the values of *T*_5%_, the thermal stabilities of the PVDF/CF composites improved slightly at 5 and 10 wt.% of the CFs. Meanwhile, the thermal stability remained almost the same, with the further addition of the CFs from 20 to 40 wt.%. On the contrary, the PVDF/SCF composites had higher decomposition temperatures, compared to the pure PVDF. At the same filler loadings, the PVDF/SCF composites became more thermally stable, compared to the PVDF/CF composites. Although the thermal stability of the PVDF/SCF composites was improved, there was a slight decrease for the PVDF/SCF composites with 5 and 10 wt.% fillers, compared to the PVDF/SCF with 20 wt.% filler. The PVDF/SCF composites also showed higher *T_max_*, and the PVDF/SCF-20 and PVDF/SCF-40 had the highest peak temperature of 450 °C, which was 25 °C higher than that of the pure PVDF. This is due to the better dispersion of the small-sized CFs into the PVDF matrix, which contributes to the enhanced thermal stability of the PVDF matrix. At higher loadings, the original CFs cannot be dispersed well in the PVDF matrix; thus, the enhancement of the thermal stability was moderate at best. For the small-sized CFs, it was easier to disperse in the PVDF matrix, even at high loadings (Figure 3).

### 3.6. Dynamic Mechanical Properties of PVDF Composites

**Figure 9:**
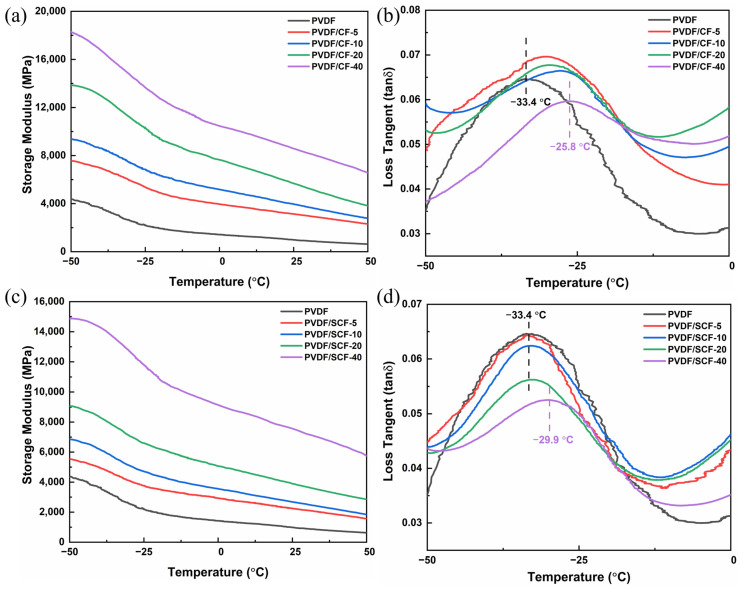
DMA test results of pure PVDF and PVDF composites (**a**,**c**): energy storage modulus of PVDF/CF and PVDF/SCF composite series, (**b**,**d**): loss factor of PVDF/CF and PVDF/SCF composite series.

The dynamic mechanical properties of the pure PVDF and PVDF composites were investigated by DMA. Figure 9 shows the temperature dependence of the storage modulus and loss factor of the samples. The storage modulus of the pure PVDF was approximately 4.3 GPa at −50 °C and decreased from −50 to 50 °C (Figure 9). The pure PVDF showed a peak at −33.4 °C, which corresponds to its glass transition temperature (*T_g_*). The *T_g_* represents the start temperature of the segmental motion of the amorphous PVDF portion [53].

The storage modulus of the PVDF/CF composites gradually increased with the CF loadings (Figure 9a). As CFs have a higher elastic modulus, adding CFs into the PVDF matrix enhanced the storage modulus of the PVDF composites. Besides, the “physical crosslinking” interaction of the CFs between the CFs and the PVDF matrix indicated the formation of the CF network structures, which promoted the load transfer between the fillers and the matrix through the network [54]. From Figure 9b, the loss factor peak of the PVDF/CF composites, induced by α relaxation [53], moved to higher temperatures, which represents the improved *T_g_* of the PVDF matrix after adding the CFs. The increased *T_g_* resulted from the restriction of CFs to the segmental motions of molecular chains of the PVDF. Consequently, more energy is required for the segmental motions of the molecular chains of the PVDF [54].

The PVDF/SCF composites demonstrated similar dynamic mechanical behaviors (Figure 9c,d). However, there were some obvious differences, due to the size effect of the fillers. Although the small-sized CFs increased the storage modulus, the increment of the PVDF/SCF composites was significantly lower than that of the PVDF/CF composites at the same filler loadings. For example, the storage modulus of the PVDF/CF-5 was up to 7600 MPa, which was 1000 MPa higher than that of the PVDF/SCF-5. This indicates that the small-sized CFs cannot transfer load from the PVDF matrix to the fillers as well as the original size CFs. Moreover, the original size CFs shifted the *T_g_* to a higher temperature region. The highest *T_g_* was up to −25.8 °C for the PVDF/CF-40. However, the small-sized CFs did not have obvious positive impacts on the *T_g_*. This is because the small-sized CFs imposed less restriction on the movement of the molecular chains of the PVDF.

### 3.7. Mechanical Properties of PVDF Composites

**Figure 10:**
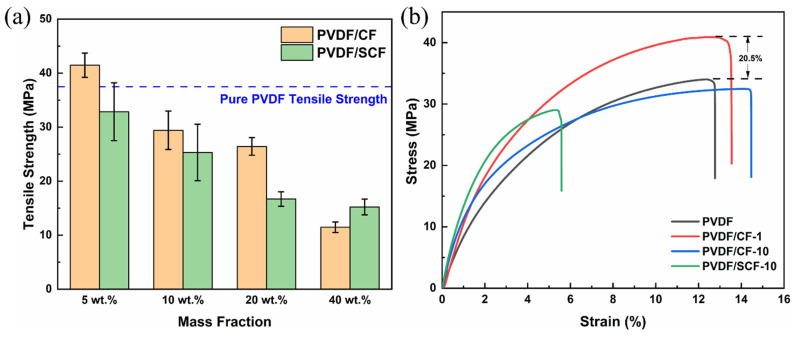
Mechanical properties of the pure PVDF and the PVDF composites: (**a**) tensile strength, (**b**) stress–strain curves.

The tensile strength of the PVDF composites is presented in Figure 10, and the PVDF/CF-5 had the highest tensile strength, up to 42.9 MPa (Figure 10a), nearly 14% higher than that of the pure PVDF. With further increasing CF loadings, the tensile strength of the PVDF/CF composites decreased significantly. The explanation is that, at low filler loadings, the dispersion of the CFs was moderately uniform, and the compatibility between the CFs and the PVDF matrix was better, which helped the external load transfer from the PVDF to the CFs. Therefore, the tensile strength of the composites was reasonably enhanced. However, the dispersion of the CFs at high loadings became poorer, compared to low loadings, and the poor interfacial adhesion caused by the inert surface of the CFs limited the load transfer from the PVDF matrix to the CFs. Therefore, the mechanical strength decreased with the further addition of the CFs. For the PVDF/SCF composites, the tensile strength decreased with increasing the SCF loadings, and the value was lower than that of the PVDF/CF composites at the same filler loadings. This is because the poorer compatibility between the SCFs and the PVDF matrix limited the load transfer and reduced the mechanical stability of the PVDF matrix. Therefore, the tensile strength of the PVDF/SCF composites declined.

Figure 10b shows the stress–strain curves of some representative samples. The initial linear slope of a stress–strain curve corresponds to the elastic modulus of a material [34]. As shown in Figure 10b, the modulus of all the PVDF composites was slightly greater than that of the pure PVDF, and a higher CF loading led to a larger tensile modulus. Meanwhile, the slope of the PVDF/SCF composites was larger than that of the PVDF/CF with the same loadings. These changes indicate that adding CFs makes the PVDF matrix more rigid. The more the CFs are added, the more brittle the PVDF composites become. The PVDF/SCF composites are more rigid than the PVDF/CF composites at the same filler loadings. In short, the higher filler loadings and shorter length gradually changed the PVDF composites from ductile to brittle modes [54].

### 3.8. Chemical Stability

**Figure 11:**
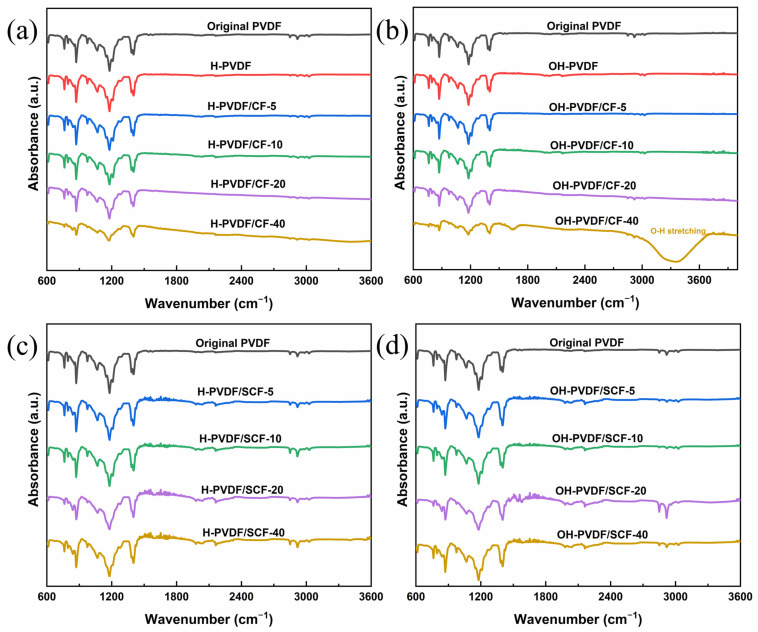
FTIR spectra of the pure PVDF and the PVDF/CF composites in (**a**) acid and (**b**) alkaline environments; and the PVDF/SCF composites in (**c**) acid and (**d**) alkaline environments.

To evaluate the chemical resistance of the PVDF composites, the samples were immersed in either 1 M of sulfuric acid solution or sodium hydroxide solution for 10 days. Figure 11 shows the FTIR spectra of the samples after immersing in acid and alkaline environments. For the pure PVDF, no obvious absorption peaks appeared in the spectra, suggesting that the pure PVDF was chemically stable in acid and alkaline environments. For the PVDF/CF composites, all the samples exhibited a good chemical resistance to acid and alkaline environments, which means that adding CFs into the PVDF matrix did not affect the chemical stability of the PVDF significantly. However, the PVDF/CF-40 showed an obvious new absorption peak at around 3500 cm^−1^, characterized as O-H stretching (Figure 11a). This may be the result of the poor compatibility and aggregation of the CFs at higher loadings. The new peak appeared due to the infiltration of water into voids between the CFs and PVDF matrix at higher CF loadings. The exposure of the CFs at the surface may lead to the formation of a weak fiber-matrix interface that is easily infiltrated by moisture; thus, the peak of water appeared. For the PVDF/SCF composites, there was no new peak in the spectra (Figure 11c,d), suggesting that the PVDF/SCF composites were chemically stable in acid and alkaline environments. In short, the PVDF-based composites showed good chemical stabilities.

## 4. Conclusions

Thermally enhanced PVDF/CF composites were successfully fabricated by cold pressing, followed by sintering. This work investigated the addition of CFs and their length effects on the thermal and mechanical properties of PVDF composites. The thermal conductivity of the PVDF significantly improved after adding CFs into the PVDF matrix. The PVDF/SCF-40 had the highest through-plane thermal conductivity, up to 2.89 W/(m∙K), with an enhancement of 1589%, compared with the pure PVDF. Compared to the original-sized CFs, the small-sized CFs not only improved the thermal conductivity of PVDF more at higher CF loadings, but also contributed to the conversion of PVDF crystalline from the α-phase to β-phase. However, there was no obvious change in the crystalline structure of the PVDF matrix. In addition, the thermal stability of the PVDF/SCF composites was better than that of the PVDF/CF composites. The highest maximum weight loss temperature of the PVDF/SCF-20 and PVDF/SCF-40 was up to 450 °C, which was 25 °C higher than that of the pure PVDF. Due to the poor compatibility between the CFs and PVDF matrix, the PVDF composites showed poor mechanical strength at high filler loadings. All the PVDF-based composites had good chemical stabilities. This study provides a simple way to largely enhance the through-plane thermal conductivity of PVDF-based composites, compared to other works. Synthesized PVDF composites can be promising materials for membrane heat exchangers. The mechanical strength of the composite reduced with the increase of the CF loading, and there should be a trade-off between the thermal transport and robustness of materials for practical applications (e.g., polymer heat exchangers). To further increase the thermal conductivity of the PVDF composites and maintain the mechanical strength, future studies on enhancing the compatibility between the CFs and PVDF matrix are needed.

## Figures and Tables

**Table 1:** Sample information of the prepared composites, in terms of filler loadings.

Samples	Fillers	Loadings (wt.%)
PVDF/CF-5	CFs	5
PVDF/CF-10		10
PVDF/CF-20		20
PVDF/CF-40		40
PVDF/SCF-5	SCFs	5
PVDF/SCF-10		10
PVDF/SCF-20		20
PVDF/SCF-40		40

**Table 2:** Percentage proportions of the α- and β-phase in the PVDF composites.

Sample	α-Phase (%)	β-Phase (%)
PVDF	65	35
PVDF/CF-5	61	38
PVDF/SCF-5	59	41

**Table 3:** Degradation properties of the pure PVDF, PVDF/CF, and PVDF/SCF composites.

Sample	Weight Loss Temperature (°C)			Residual (%)
	*T*_5%_	*T*_10%_	*T*_*max*_	
PVDF	390	398	425	33.5
PVDF/CF-5	407	414	432	36.5
PVDF/CF-10	403	412	429	40.4
PVDF/CF-20	393	403	432	43.2
PVDF/CF-40	391	399	396	58.4
PVDF/SCF-5	414	421	440	36.8
PVDF/SCF-10	404	412	428	40.3
PVDF/SCF-20	427	435	450	41.6
PVDF/SCF-40	424	437	450	54.1

## Data Availability

The data presented in this study are available on request from the corresponding author.
